# Editorial: Interpretable predictive analytics for precision cardio-oncology preventive care

**DOI:** 10.3389/fcvm.2024.1377749

**Published:** 2024-03-06

**Authors:** Jiandong Zhou, Tong Liu, Leonardo Roever, Qingpeng Zhang

**Affiliations:** ^1^ Department of Family Medicine and Primary Care, Li Ka Shing Faculty of Medicine, The University of Hong Kong, Hong Kong, Hong Kong SAR, China; ^2^ School of Public Health, Li Ka Shing Faculty of Medicine, The University of Hong Kong, Hong Kong, Hong Kong SAR, China; ^3^ Department of Pharmacology and Pharmacy, The University of Hong Kong, Hong Kong, Hong Kong SAR, China; ^4^ Division of Health Science, Warwick Medical School, University of Warwick, Coventry, United Kingdom; ^5^ Tianjin Key Laboratory of Ionic-Molecular Function of Cardiovascular Disease, Department of Cardiology, Tianjin Institute of Cardiology, Second Hospital of Tianjin Medical University, Tianjin, China; ^6^ Lebanese American University, Gilbert and Rose-Marie Chagoury School of Medicine, Lebanese American University, Beirut, Lebanon; ^7^ Musketeers Foundation Institute of Data Science, The University of Hong Kong, Hong Kong, Hong Kong SAR, China

**Keywords:** cardio-oncology, cardiovascular diseases, computational cancer therapy, biomedical big data analytics, interpretable machine learning

**Editorial on the Research Topic** Interpretable predictive analytics for precision cardio-oncology preventive care

Cardiovascular disease is a leading cause of mortality among cancer survivors globally, exceeding 370,000 deaths annually, followed by the risk of new tumour development (1). The field of cardio-oncology has emerged due to heightened awareness of cardiac dysfunction resulting from cancer therapies like chemotherapy, radiotherapy, and immunotherapy. Precision cardio-oncology emphasizes urgent early detection, monitoring, prevention, and treatment of cardiovascular toxicity associated with cancer therapies (2). Individualized algorithms, rooted in cardiovascular risk, cancer characteristics, and treatment risks, are crucial in refining tumor classification and guiding cancer treatment (3). Artificial intelligence (AI)-based predictive analytics, including supervised and unsupervised learning, have recently become instrumental in capturing intricate patterns between cardiovascular diseases and cancer therapies, offering transformative insights for personalized clinical decisions in cancer treatment within a methodologically explainable framework (4, 5).

The first article, “*Early detection of immune checkpoint inhibitor-related subclinical cardiotoxicity: A pilot study by using speckle tracking imaging and three-dimensional echocardiography*”. Xu et al., was motivated that early detection of subclinical cardiotoxicity of immune checkpoint inhibitor therapy can be challenging, and aimed to identify the subclinical cardiotoxicity associated with immune checkpoint inhibitor therapy. The study employs two-dimensional speckle tracking imaging (2D-STI) and three-dimensional echocardiography to evaluate subclinical cardiac dysfunction in Chinese patients. Results indicate that PD1/PDL1 immunotherapy may lead to impaired left and right ventricular systolic function, with LVGLS, RVGLS, and TAPSE identified as more sensitive indices for early detection of subclinical cardiotoxicity.

In the second paper is a study protocol, “*Development of an interpretable machine learning-based intelligent system of exercise prescription for cardio-oncology preventive care: A study protocol*”, Gao et al., aimed to develop an interpretable machine learning-based intelligent system of exercise prescription for cardio-oncology preventive care by recruiting people living with cancer (PLWC). Their study will develop not only an interpretable machine learning model to recommend exercise prescription but also an intelligent system of exercise prescription for precision cardio-oncology preventive care.

In the third article, “*Risk factors, prediction model, and prognosis analysis of myocardial injury after acute upper gastrointestinal bleeding*”, Hao et al. aimed to analyse the risk factors for myocardial injury in acute upper gastrointestinal bleeding (AUGIB) patients, predict the risk of myocardial injury, and explore the clinical prognosis and influencing factors in AUGIB patients with myocardial injury. This is because cardiovascular complications in patients with AUGIB have been associated with a high-risk of subsequent adverse consequences. Their findings suggest that Hypertension, renal dysfunction, and cardiac function with LVEF <68% strongly predicted myocardial injury, and that coagulopathy correlated with poor prognosis in AUGIB patients with myocardial injury, emphasizing critical implications for clinical management.

The fourth article, “*Long-term cardiovascular mortality risk in patients with bladder cancer: a real-world retrospective study of 129,765 cases based on the SEER database*”, authored by Liao et al., aimed to identify the independent risk factors and develop a novel nomogram for predicting long-term cardiovascular mortality in patients with bladder cancer using data extracted from the Surveillance, Epidemiology, and End Results (SEER) database. Their study developed a novel nomogram for predicting long-term cardiovascular mortality in patients with BC based on the competing risk model. The findings could help clinicians comprehensively and effectively manage the co-patient of bladder cancer and cardiovascular diseases, thereby reducing the risk of cardiovascular mortality in bladder cancer survivors.

In the firth paper, “*Visual analysis based on CiteSpace software: a bibliometric study of atrial myxoma*”, Gao et al., seeks to use CiteSpace and VOSviewer visual metrology to analyse the research status, frontier hotspots, and trends in research on atrial myxoma over the past 20 years, by drawing a co-occurrence network, co-polymerization class, and burst terms, and a corresponding visual atlas. Their study revealed that the main research topics and hotspots in atrial myxoma included surgical methods, case reports, genetic and molecular studies.

Collectively, these articles illuminate diverse aspects of cardiovascular health and cancer, offering valuable insights for precision medicine and preventive care. These diverse findings collectively advance our understanding of cardiovascular complications, precision medicine, and holistic care strategies, shaping new directions for future research and clinical applications in cardio-oncology.

By bringing these disparate but interconnected topics under the aegis of network analysis, we aim to inspire more innovative multidisciplinary methodologies enhancing interpretability in AI predictions, validating diagnostic, prognostic, and therapeutic models for cardiac dysfunctions during and after cancer therapies. Additionally, the focus extends to translational medical implications, exploring automatic data processing techniques for handling massive longitudinal/multimodal data in cardio-oncology and developing advanced workflows for innovative pattern discovery, visualization, risk predictions, and treatment recommendations in cardio-oncology care.
